# S04-5: Lessons learned from the results of policy audits of multiple local governments

**DOI:** 10.1093/eurpub/ckae114.215

**Published:** 2024-09-26

**Authors:** Noriko Takeda, Yukio Oida, Shigeru Inoue, Motohiko Miyachi

**Affiliations:** Kogakuin University, Tokyo, Japan; Chukyo University, Aichi, Japan; Tokyo Medical University, Tokyo, Japan; Waseda University, Saitama, Japan

## Abstract

**Purpose:**

To promote health-enhancing physical activity (HEPA), it is essential to evaluate physical activity policies not only at the national level but also the local level. We have conducted research on physical activity policies in multiple local governments across Japan.

**Methods:**

Japan has 47 prefectures and 1,718 municipalities. We first developed a Local Policy Audit Tool (L-PAT) for prefectures, comprising 11 items based on WHO’s HEPA Policy Audit Tool (PAT) that cover key policy areas. We then developed a City PAT (C-PAT) with six questions based on the HEPA PAT. All 47 prefectures were targeted for the L-PAT study, and 272 municipalities were targeted with the C-PAT, randomly selected by municipality size. For both tools, we asked key government departments (e.g. health, sports, urban planning and transport) for their responses.

**Results:**

The response rate to the L-PAT was 71.6%. Results indicate that health and sports are the most active departments in promoting HEPA at a prefecture level. Some prefectures also reported actions by the urban planning and transport departments. The response to the C-PAT was 37.7%. The rate of policy formulation was extremely high in the health and sports departments, with slight variation by population size. By contrast, policy formulation rates were generally low in the urban planning, transport and environment departments, especially in smaller municipalities.

**Conclusions:**

The advantage of the L-PAT and C-PAT is that they can quantitatively assess the status of local government HEPA policies. This allows for comparisons by state, by population size, and between countries. The assessment at the municipal level showed the impact of the size of the municipality. This may help us to provide better support to these local governments from the national government. The next steps could be to revise the questionnaires to increase their usefulness, to consider ways to increase the response rate, and to use them for continuously monitoring local HEPA policies.

